# Phosphate of Lime in Scrofula, &c.

**Published:** 1852-03

**Authors:** 


### *Phosphate of Lime in Scrofula and other Depraved States of the System.* By M. Beneke.

[M. Beneke has contributed two papers on this subject to the London Lancet, for July and September, 1851. Not having space for the whole, we present our readers with the resume.]

To sum up now the results of the first and second part of these communications, I have shown in the first that, supposing a sufficient quantity of albumen and fat to be present, the produce of cells evidently increases by the administration of phosphate of lime; that, on the other hand, by this administration we may promote the cure of diseases which show a deficiency of formation of cells; and that especially in scrofulous affections the administration of phosphate of lime has often proved most beneficial. On the other hand, in the second part, I have established the fact, that in nearly all chronic diseases, where we observe a loss of flesh, emaciation, and general weakness, a hypernormal quantity of phosphates is always excreted from the economy by the urine, and more especially in those cases where the administration of phosphate of lime proved most beneficial. Perhaps it might be supposed that these quantities had been increased by the phosphate of lime taken as a remedy; but this is by no means the case; on the contrary, my observations prove that, even during the administration of phosphate of lime, the quantity of earthy phosphates in the urine often decreases, supposing a proper treatment in other respects to be employed. Well, then, the harmony of the results of the above two parts is so striking, that we scarcely can admit of any doubt in their truth, and the physiological as well as the pathological importance of the points alluded to is so apparent, that it does not require any further explanation. We know that the phosphate of lime is indispensably necessary for the production of cells; we know that in a great many diseases the phosphates are excreted from the economy in very abundant quantities by the urine; and we know even that in these diseases the formation of cells is deficient. Shall we have any doubt that by substituting the quantity of phosphates excreted by the urine, or by removing the cause of their excretion itself, we must afford a great benefit to persons who are afflicted with the diseases alluded to? * — *Charleston Medical Journal and Review.*

---

* In the October number of the N. O. Monthly Medical Register Dr. Stone has added his testimony to that of M. Beneke, in reference to the beneficial effects of this salt in tuberculosis and scrofulosis. It has been also employed in cases of caries, fracture, &c., occurring especially in strumous constitutions.—Eds. Review.

